# Culture Model of Rat Portal Myofibroblasts

**DOI:** 10.3389/fphys.2016.00120

**Published:** 2016-03-31

**Authors:** Haquima El Mourabit, Emilien Loeuillard, Sara Lemoinne, Axelle Cadoret, Chantal Housset

**Affiliations:** ^1^Centre de Recherche Saint-Antoine, Institute of Cardiometabolism and Nutrition, Sorbonne Universités, UPMC Univ Paris 06, INSERM, UMR_S 938Paris, France; ^2^Assistance Publique-Hôpitaux de Paris, Hôpital Saint-Antoine, Centre de Référence Maladies Rares des Maladies Inflammatoires des Voies Biliaires, Service d'HépatologieParis, France

**Keywords:** alpha-smooth muscle actin, bile ducts, collagen-type XV-alpha 1, cytokeratin 19, fibulin 2, liver fibrosis, liver digestion, portal tract

## Abstract

Myofibroblasts are matrix-producing cells with contractile properties, usually characterized by *de novo* expression of alpha-smooth muscle actin, that arise in fibrotic diseases. Hepatic stellate cells (HSCs), known as perisinusoidal cells containing auto-fluorescent vitamin A, are the major although not exclusive source of myofibroblasts in the injured liver. Portal myofibroblasts (PMFs) have been defined as liver myofibroblasts derived from cells that are distinct from HSCs and located in the portal tract. Here, we describe the protocol we have established to obtain rat PMFs in culture. In this method, the biliary tree is (i) separated from the liver parenchyma by *in situ* enzymatic perfusion of the liver, (ii) minced and further digested *in vitro*, until bile duct segments are isolated by sequential filtration. Bile duct isolates free of HSC contaminants, form small cell clusters, which initially comprise a large majority of epithelial cells. In culture conditions (fetal bovine serum) that provide a growth advantage to mesenchymal cells over epithelial cells, the epithelial cells die and detach from the substrate, while spindle-shaped cells outgrow from the periphery of the cell clusters, as shown by video-microscopy. These cells are highly proliferative and after 4–5 days, the culture is composed exclusively of fully differentiated myofibroblasts, which express alpha-smooth muscle actin and collagen 1, and secrete abundant collagen. We found no evidence for epithelial-mesenchymal transition, i.e., no co-expression of alpha-smooth muscle actin and cytokeratin at any stage, while cytokeratin becomes undetectable in the confluent cells. PMFs obtained by this method express the genes that were previously reported to be overexpressed in non-HSC or portal fibroblast-derived liver myofibroblasts as compared to HSC-derived myofibroblasts, including the most discriminant, collagen 15, fibulin 2, and Thy-1. After one passage, PMFs retain the same phenotypic features as in primary culture. In conclusion, this straightforward and reproducible method of PMF culture, can be used to identify new markers of PMFs at different stages of differentiation, to compare their phenotype with those of HSC-MFs and ultimately determine their progenitors and specific functions in liver wound-healing.

## Introduction

Myofibroblasts are cells that arise in fibrotic diseases. They are matrix-producing cells with contractile properties, characterized by *de novo* expression of proteins shared with smooth muscle cells. Myofibroblasts form a heterogeneous population of cells with different possible origins. Alpha-smooth muscle actin (α-SMA) is their most commonly used marker (Hinz et al., 2012). Hepatic stellate cells (HSCs) have been identified as the major source of myofibroblasts in the injured liver (Friedman, 2008), in particular by studies of cell lineage tracing (Mederacke et al., 2013). HSCs are recognized in their quiescent state, as vitamin A-storing cells located in the perisinusoidal space of Disse. However, culture studies and *in situ* studies of fibrotic livers have provided evidence to indicate that liver myofibroblasts could also derive from cells that are distinct from HSCs and located in the portal area (Cassiman et al., 2002; Uchio et al., 2002; Kinnman et al., 2003; Beaussier et al., 2007; Li et al., 2007). Today, we refer to this sub-population of liver myofibroblasts as portal myofibroblasts (PMFs) (Lemoinne et al., 2013). Cells called portal fibroblasts have also been described. They are periductal α-SMA-negative fibroblastic cells found in the normal liver, that express ecto-nucleoside triphosphate diphosphohydrolase 2 (ENTPD2) (Tuchweber et al., 1996; Dranoff et al., 2002). There is evidence to indicate that portal fibroblasts can become activated, i.e., myofibroblastic, in biliary-type liver fibrosis. However, this does not mean that PMFs all derive from portal fibroblasts. In fact, the progenitor cells of PMFs have not been identified yet. To a large extent, our knowledge regarding PMFs is based on a rat culture model that we previously established (Kinnman et al., 2003; Bosselut et al., 2010). In this model, PMFs are obtained by outgrowth from bile duct preparations. We showed that PMFs obtained by this method expressed a number of genes at higher levels than HSC-derived myofibroblasts (HSC-MFs) (Lemoinne et al., 2015). This included fibulin 2 and other genes such as Thy-1, gremlin 1, and fibronectin 1 that were also found by others to be overexpressed in non-HSC derived liver myofibroblasts as compared to HSC-MFs (Knittel et al., 1999; Ogawa et al., 2007; Dudas et al., 2009). This also included the most discriminant marker of non-HSC derived myofibroblasts identified so far, with the highest expression ratio relative to HSC-MFs, i.e., collagen-type XV-alpha 1 (COL15A1).

Non-HSC derived liver myofibroblasts were previously obtained in culture, by different methods of cell isolation. The so-called rat liver myofibroblasts were isolated by enzymatic digestion of the liver, followed by separation of non-parenchymal cells by density gradient and purification of a fraction enriched in myofibroblast precursors by elutriation (Knittel et al., 1999). Vitamin A-free cells were also isolated from a stellate cell-enriched fraction of normal rat liver by fluorescence-activated cell sorting (FACS) (Ogawa et al., 2007). Cells that were negative for ultraviolet-autofluorescence of vitamin A were thus obtained by FACS and formed myofibroblasts in culture, with distinctive features compared to HSC-MFs. Another method has been described (Kruglov et al., 2002) and subsequently modified (Wen et al., 2012), to isolate cells assumed to be portal fibroblasts, from rat liver. First, the biliary tree is prepared by enzymatic digestion of the liver and isolated cells presumably enriched in portal fibroblasts, obtained by size-based filtration. Two markers of portal fibroblasts have been reported, ENTPD2, which is lost after myofibroblastic differentiation in culture, and elastin, which is maintained.

None of these methods, has yet allowed to clearly identify the progenitor cells distinct from HSCs that contribute to liver myofibroblasts, their fate and functions, compared to those of HSC-MFs in liver tissue repair. The advantage of our model is that (i) the outgrowth of myofibroblasts reproduces the pattern of fibrosis progression from the portal area toward the lobule observed *in vivo*; (ii) portal progenitors are not dissociated from their initial niche, avoiding cell selections; (iii) the protocol, phenotype and markers of myofibroblasts obtained by this method, are all very reproducible. The limitations are the abundance of contaminant bile duct epithelial cells in the initial preparation, and possibly, a diversity of myofibroblast progenitor cells. This protocol can be used to identify new markers of PMFs at different stages of differentiation, to compare the behavior and functions of these cells with those of HSC-MFs and determine their interactions with HSC-MFs.

## Materials and equipment

*N.B*. European catalog numbers (Cat. No.) are provided.

### Animals

Sprague Dawley rats weighing 150–200 g (Janvier Labs). Different strains (e.g., Wistar) can be used. The protocol is optimal when rats body weight is ≤250 g. Animals were housed under specific pathogen free conditions (PHEA, agreement No: B 75-12-01).

### Enzymes/chemicals

- Betadin®
- Bovine serum albumin (BSA, Sigma-Aldrich Cat. No. A7030)
- Collagenase P (Sigma-Aldrich Cat. No. 11 213 873 001)
- DNAse (Sigma-Aldrich Cat. No. DN25 > 400 units/mg)
- EDTA 0.5 mol/L pH 8.0 (Sigma-Aldrich Cat. No. 03690)
- Hyaluronidase (Sigma-Aldrich Cat. No. H3884 type IV-S750 3000 units/mg)
- Pronase (Roche Cat. No. 11 459 643 001)
- Sodium heparin (5000 IU/ml)
- Sodium pentobarbital (CEVA animal healthy 5.47 g/100 ml)

### Culture media

- DMEM (Sigma-Aldrich Cat. No. D6046)
- Fetal Bovine Serum (FBS, GIBCO Invitrogen Cat. No. 10270-098)
- HBSS with MgCl2 and CaCl2 (GIBCO Invitrogen Cat. No. 24020-022)
- HBSS without MgCl2 or CaCl2 (GIBCO Invitrogen Cat. No. 14170-088)
- Hepes (GIBCO Invitrogen Cat. No. 15630)
- L15 Leibovitz medium (Sigma-Aldrich Cat. No. L5520)
- MEM with Earl's without glutamin (GIBCO Invitrogen Cat. No. 21090-022)
- NaCl solution (9 g/L) (OTEC Cat. No. 600502)
- Penicillin/streptomycin (GIBCO Invitrogen Cat. No. 15140-122)

### Equipment

- 12-well plates
- 16 gauge X 50 mm IV catheter (Jelco Cat. No. 4012)
- Bottle-top filter 0.22 μm (Thermo scientific Cat. No. 568-0020)
- Bubble trap (Medi-Globe Cat. No. 200927)
- Cell strainer 40 and 100 μm Nylon (Falcon Cat. Nos. 352340 and 352360)
- Cotton and linen threads
- Falcon tubes 50 mL
- Glass beakers 250 mL
- Heat lamp
- Orbital water bath
- Perfusion line (Masterflex Cat. No. 96410-16)
- Perfusion pump (Masterflex Cat. No. 751800)
- Plate with magnetic stirrer
- Refrigerated (+4°C) bench-top centrifuge
- Schott Pyrex bottles 150, 250, and 500 mL
- Sterile filter for syringe (0.22 μm)
- Sterile Petri dishes
- Sterile pipettes 5, 10, 25 mL
- Sterilized fork and scissors
- Syringes 3 and 50 mL
- Water bath

### Reagent set-up

- Dilute pentobarbital at 1/10 in NaCl solution.
- EDTA solution (19 mg/L): Prepare the solution by dissolving 6.5 mL EDTA in 43.5 mL sterile water and filter through a 0.22-μm syringe filter. This solution can be prepared up to 4 weeks in advance and stored at +4°C.
- L15 1% Hepes solution: Under sterile condition, dilute 5 mL Hepes (1 mol/L) in 500 mL L15 medium.
- Wash solution (HBSS without MgCl2 or CaCl2, 1% EDTA): Dilute 2 mL of 19-mg/L EDTA solution in 200 mL HBSS without CaCl2 or MgCl2. Adjust the pH to 7.35–7.40 and filter it through 0.22-μm bottle-top filter.
- Enzyme solution 1: Add 15 mg collagenase P in 200 mL HBSS with MgCl2 and CaCl2. Place the mixture on a magnetic shaker until dissolution and adjust the pH to 7.35–7.40 and filter it through 0.22-μm bottle-top filter.
- Enzyme solution 2. In 200 mL MEM, dissolve 15 mg collagenase P, 140 mg pronase, 13 mg DNAse and 200 mg BSA. Supplement with 1% penicillin/streptomycin, 1% Hepes and 3% FBS. Place the mixture on a magnetic shaker until dissolution, adjust the pH to 7.35–7.40 and filter it through 0.22-μm bottle-top filter.
- Enzyme solution 3. In 100 mL MEM, dissolve 6 mg collagenase P, 20 mg hyaluronidase, 6 mg DNAse and 100 mg BSA. Supplement with 1% penicillin/streptomycin, 1% Hepes and 3% FBS. Place the mixture on a magnetic shaker until dissolution, adjust the pH to 7.35–7.40 and filter it through 0.22-μm bottle-top filter.
- Complete DMEM: Supplement DMEM with 1% penicillin/streptomycin, 1% Hepes and 10% FBS.

▴ CRITICAL Wash and enzyme solutions should be prepared no later than 18 h before starting the isolation procedure.

### Equipment set-up

Fill perfusion line and bubble trap with wash solution and adjust the flow rate of perfusion pump at 10 mL/min.

## Procedure

### Procedure set-up (60 min)

Described in Section Reagent Set-Up and Equipment Set-Up.

▴ CRITICAL Wash solution and enzyme solution 1 should be preheated in the water bath (38°C).

### *In situ* digestion of rat liver (90 min)

**Figure 1:**
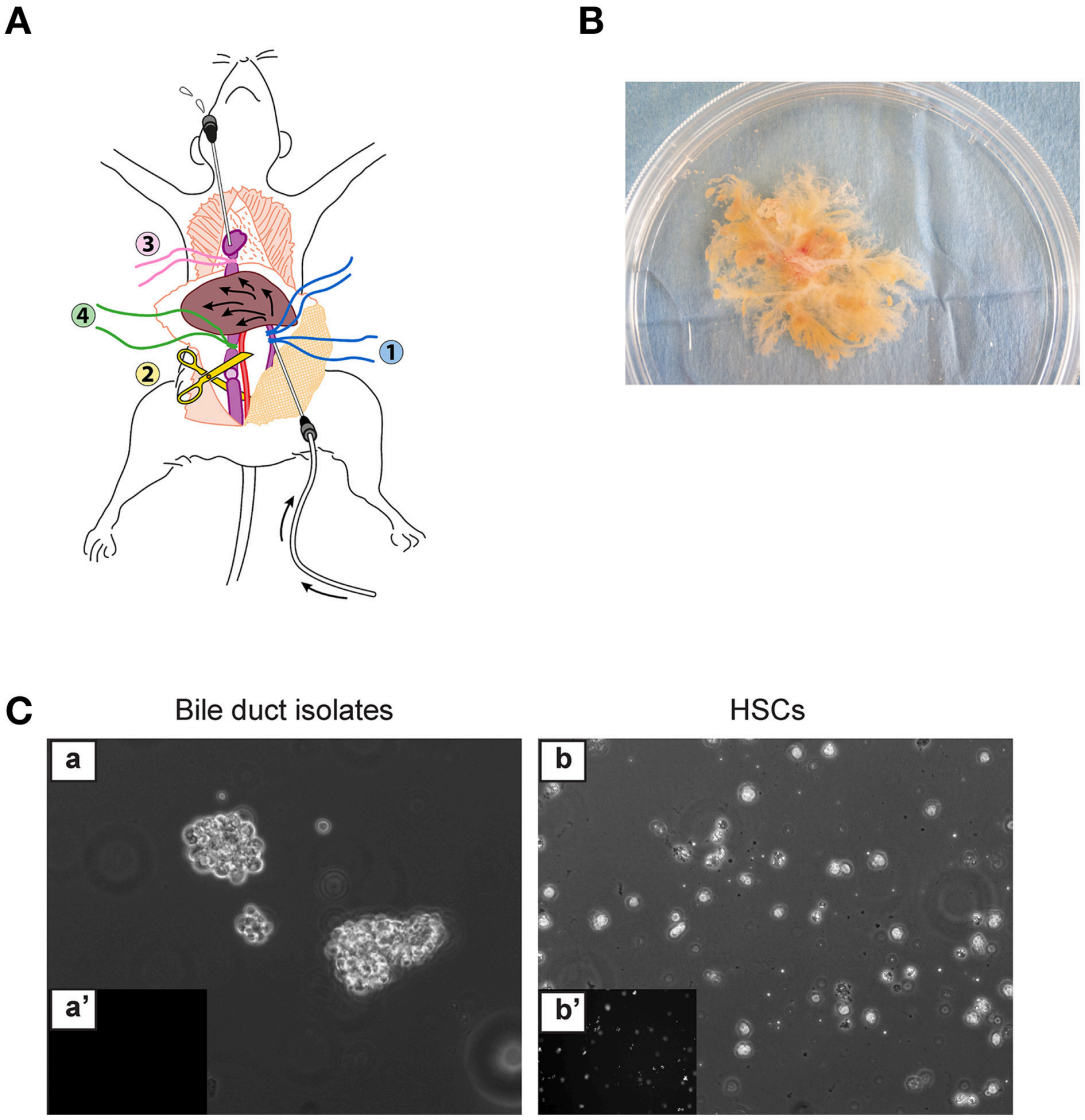
**Enzymatic digestion of the liver. (A)** Set-up for *in situ* liver perfusion. A catheter is inserted into the portal vein (blue threads) and connected to the perfusion tubing. After starting the perfusion, the vena cava is sectioned under the liver to prevent hyper-pressure in the liver. Then, a catheter is inserted through the right auricle into the inferior vena cava (pink thread) to drain the outflow from the liver, and the thread (green) around the vena cava, just above section, is ligated. **(B)** Biliary tree isolated by *in situ* digestion, placed in a 100-mm culture dish. **(C)** Bile duct isolates obtained immediately after *in vitro* digestion of the biliary tree (a,a'), in comparison with freshly isolated HSCs (b,b'), under phase-contrast microscopy (a,b) or epifluorescence microscopy at 328-nm UV excitation (a',b'), form small cell clusters free of autofluorescent HSCs.

1- Anesthetize the rat according to the institutional approved animal protocol. We perform pentobarbital intra-peritoneal injection (1 mL/100 g body weight).
2- Shave the abdomen and clean with Betadin®.
3- Immobilize the rat by the upper and lower extremities on a dissecting board.
4- Perform a laparatomy to expose the liver, and move the visceral organs to the right side to expose the inferior vena cava and the portal vein.
5- Ligate the gastro-duodenal vein with a cotton thread. Then, prepare two linen threads around the portal vein under the gastro-duodenal vein, not to ligate at this stage. Prepare also a linen thread around the infrahepatic vena cava above the renal vein, not to ligate at this stage.
6- Inject 500 IU heparin in the femoral vein.
7- Place a 16-gauge catheter into the portal vein, entering under the gastro-duodenal vein. Check the position of the catheter that should not point upward in the vein, and ligate the two linen threads around the catheter placed in the portal vein. **!** Troubleshooting (Table 1).
8- Start the pump, connect the catheter to the perfusion line and stabilize the set-up to avoid exit of the catheter. ▴ CRITICAL STEP Before connecting the perfusion line, make certain that no bubbles are introduced in the catheter, which would result in improper digestion.
9- Section the infrahepatic vena cava beneath the unligated linen thread that was prepared in step 5.
10- Place a 16-gauge catheter through the right auricle into the suprahepatic vena cava and fix it with a linen thread.
11- Ligate the infrahepatic vena cava by using the linen thread that was prepared in step 5. Thereafter, perfused solutions will outflow through the auricle catheter (Figure 1A).
12- Perfuse the liver with wash solution for a total of 20 min. Switch on the heat lamp directed toward the rat liver to maintain a temperature of 37°C and hydrate the liver with NaCl solution during perfusion. ▴ CRITICAL STEP Make sure that all liver lobules are perfused. If it was not the case, you can gently move the catheter.
13- Switch from wash solution to enzyme solution 1 and perfuse the liver for 15–20 min, until digestion becomes visible.
14- Disconnect the perfusion line from the catheter but do not remove the intra-portal catheter from the liver. Explant the liver and place it in a sterile 100-mm Petri dish. Inject 20 mL of cold L15 medium (+4°C) with a syringe through catheter to dislocate hepatocytes, and repeat this injection twice. **!** Troubleshooting (Table 1).
15- Under cell culture hood, transfer the liver to a new sterile 100-mm Petri dish with L15 medium at room temperature. Peel off the liver capsule and detach the liver parenchyma by scrubbing with a fork, until the biliary tree is isolated (Figure 1B).

**Table 1:** **Troubleshooting**.

**Step**	**Problem**	**Possible reason**	**Solution**
7	Catheter exit from the portal vein	Abrupt manipulation	Inverse perfusion direction by placing the catheter in the right auricle and use it for perfusion; linen threads around the portal vein are removed to allow outflow
14	Catheter exit from the explanted liver	Abrupt manipulation	Inject L15 medium through the liver capsule using a syringe
23	Tan color of hepatocytes visible in preparations remaining on the 40-μm cell strainer	Contamination during filtration steps	Increase the number of filtration through 40-μm cell strainers
25	Low purity (contaminating hepatocytes)	Incomplete digestion	Make sure that the enzyme concentrations are correct Make sure that all liver lobules are perfused

### *In vitro* biliary tree digestion (150 min)

▴ CRITICAL Enzyme solutions 2 and 3 should be preheated in the water bath (38°C).

16- Transfer the biliary tree to a 100-mm sterile Petri dish containing pre-warmed enzyme solution 2. Cut the biliary tree into small pieces with scissors and transfer the minced biliary tree into a 250-mL Schott Pyrex bottle. Add enzyme solution 2 to a final volume of 100 mL and place the bottle in an orbital water bath at 37°C. Shake the bottle during 30 min. ▴ CRITICAL STEP The result of mincing should be a homogeneous preparation of very small fragments.
17- Filter this preparation through a 100-μm cell strainer and then through a 40-μm cell strainer. Discard the filtrate from the 40-μm cell strainer.
18- Suspend the preparations remaining on the 100-μm and 40-μm cell strainers in 100 mL of enzyme solution 2. Place the mixture under shaking at 37°C, for 30 min. ▴ CRITICAL STEP Increase the digestion time if chunks are visible.
19- Repeat step 17.
20- Suspend the preparations remaining on the 100 and 40-μm cell strainers in 100 mL of enzyme solution 3. Place the mixture under shaking at 37°C for 30 min. ▴ CRITICAL STEP Exceeding 40 min of incubation may result in a poor yield of bile duct segments.
21- Filter the mixture through a 100-μm cell strainer and collect the filtrate for two additional filtrations, each time with a new 100-μm cell strainer.
22- Subject the filtrate obtained in step 21 to filtration through a 40-μm cell strainer. Discard the filtrate.
23- Suspend the preparation remaining on the 40-μm cell strainer in L15 medium. Proceed to a last filtration through a 40-μm cell strainer and discard the filtrate. **!** Troubleshooting (Table 1).
24- Suspend the preparation remaining on the 40-μm cell strainer in 50 mL complete DMEM and transfer it into a 50-mL Falcon tube.

### Cell culture

25- Centrifuge at 1900 rpm during 8 min at room temperature. Discard the supernatant. Suspend the cell pellet in complete DMEM and plate the cells in 12-well culture dishes (1 mL/well). The yield of cell clusters is 7000–10,000 per rat liver. ▴ CRITICAL STEP The volume of complete DMEM should be adapted to the size of the cell pellet so as to obtain four to six cell clusters per microscope field at magnification x10. Perform microscope observation under 328-nm UV excitation to exclude contamination by HSCs (Figure 1C). **!** Troubleshooting (Table 1).
26- Maintain cell culture at 37°C in a 5% CO_2_ incubator. Replace the culture medium 24 h after seeding and every 48 h, thereafter. Generally, cells are confluent after 4–5 days in primary culture, a stage we refer to as passage 0 (P0).
27- Confluent cells can undergo several passages. They are fully differentiated in myofibroblasts, in primary culture (P0) and after one passage (P1).

## Results

### Methods used to characterize PMF culture

#### RT-PCR

Total RNA was used to prepare cDNA. Quantitative real-time PCR was performed using Sybr Green Master Mix on a Lightcycler 96 (Roche). Target gene mRNA levels were reported relative to a calibrator according to the 2^−ΔΔCt^ method with hypoxanthine guanine phosphoribosyl transferase (*Hprt*) used as the reference gene. Primer sequences are provided in Supplementary Table 1.

#### Fluorescence

To monitor cell viability in culture, NucGreen® Dead 488 reagent (ThermoFisher) was added to the culture medium, following the manufacturer's instructions. For immunofluorescence, cell preparations were fixed in 4% paraformaldehyde for 15 min, then blocked and permeabilized, by incubation in 2% albumin (Roche) supplemented with 0.1% Triton X100 (Sigma-Aldrich) for 1 h. Fixed cells were incubated with the primary antibodies against: COL15A1 (HPA017913, 1/30, Sigma-Aldrich), α-SMA (1A4, 1/100, Dako), or pan-cytokeratin (sc-8018, 1/10, Santa Cruz Biotechnology) overnight at 4°C. Primary antibody was revealed by Alexa Fluor 488 or 568-conjugated antibodies (1/200, Life Technologies) and DAPI was used for nuclear staining. For dual α-SMA and pan-cytokeratin immunofluorescence, Alexa Fluor 488-conjugated α-SMA (1/100, Abcam) and Alexa Fluor 647-conjugated pan-CK (1/10, Cell Signaling) antibodies were used. Image acquisition was performed, using a SP8 confocal microscope (Leica). For proliferation assay, fixed cells were incubated with the Alexa Fluor 488-conjugated Ki67 antibody (1/50, Cell Signaling) overnight at +4°C and DAPI was used for nuclear staining. Ki67-positive cells was counted in 5 random fields at magnification x20 using ImageJ software and reported to the total cell numbers (%).

#### Collagen assay

Conditioned medium prepared from cells incubated with or without 10% FBS for the last 24 h, was analyzed for soluble collagen using the Sircol collagen assay, according to the manufacturer (Sigma-Aldrich). Briefly, Sircol reagent was added to the conditioned medium to form collagen-dye complex. The precipitates were collected by centrifugation, dissolved in 0.5 mol/L NaOH and dye concentration was estimated by spectrophotometry at 540 nm.

### PMF culture characterization

**Figure 2:**
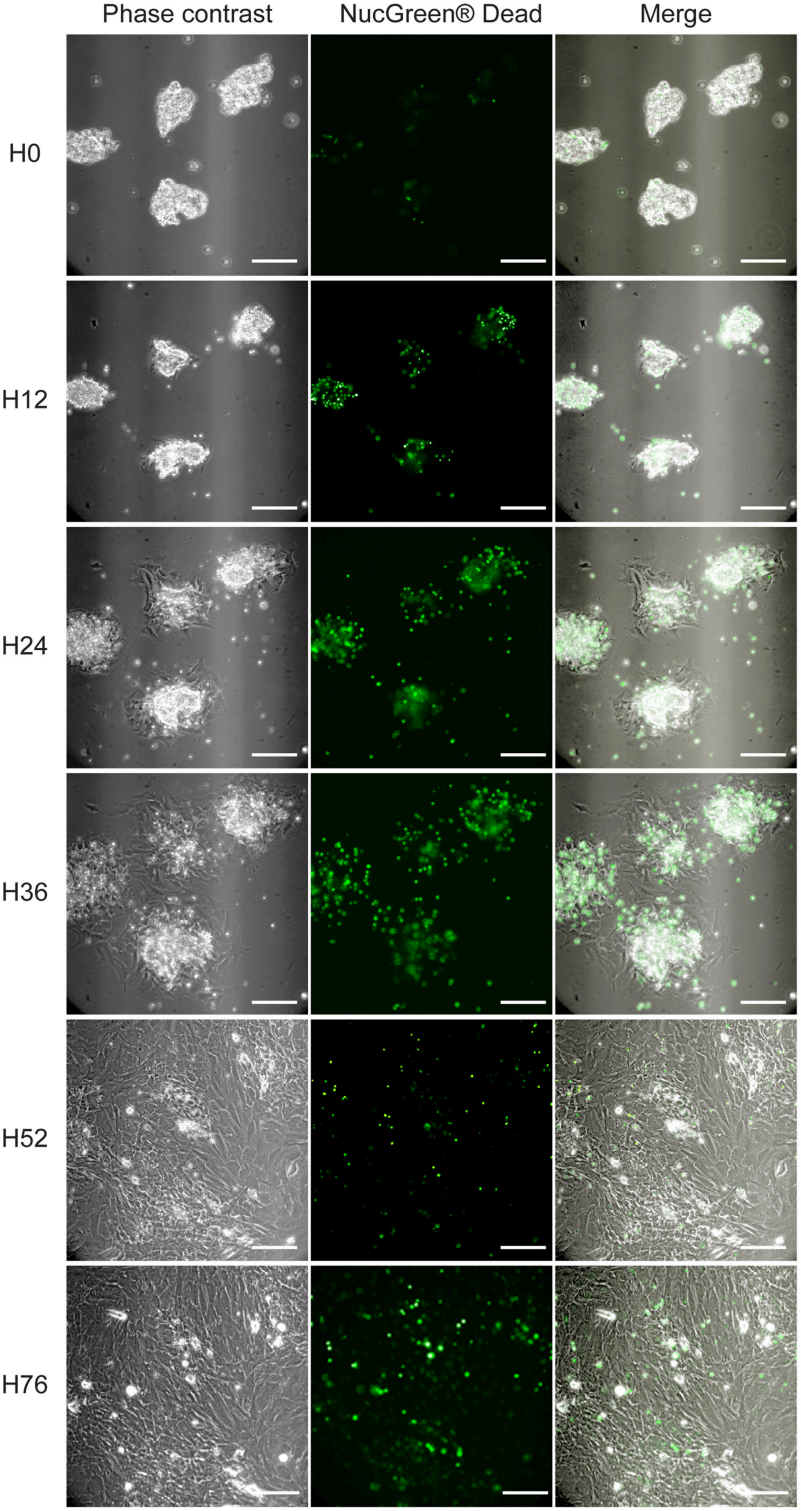
**PMF outgrowth in culture**. PMF culture in complete medium (i.e., supplemented with 10% FBS), containing NucGreen® Dead reagent to monitor cell death, was observed by Video-Microscopy (Supplementary Video). Phase-contrast, fluorescent and merge images at different time points are shown. H, hours; Scale bars, 50 μm.

**Figure 3:**
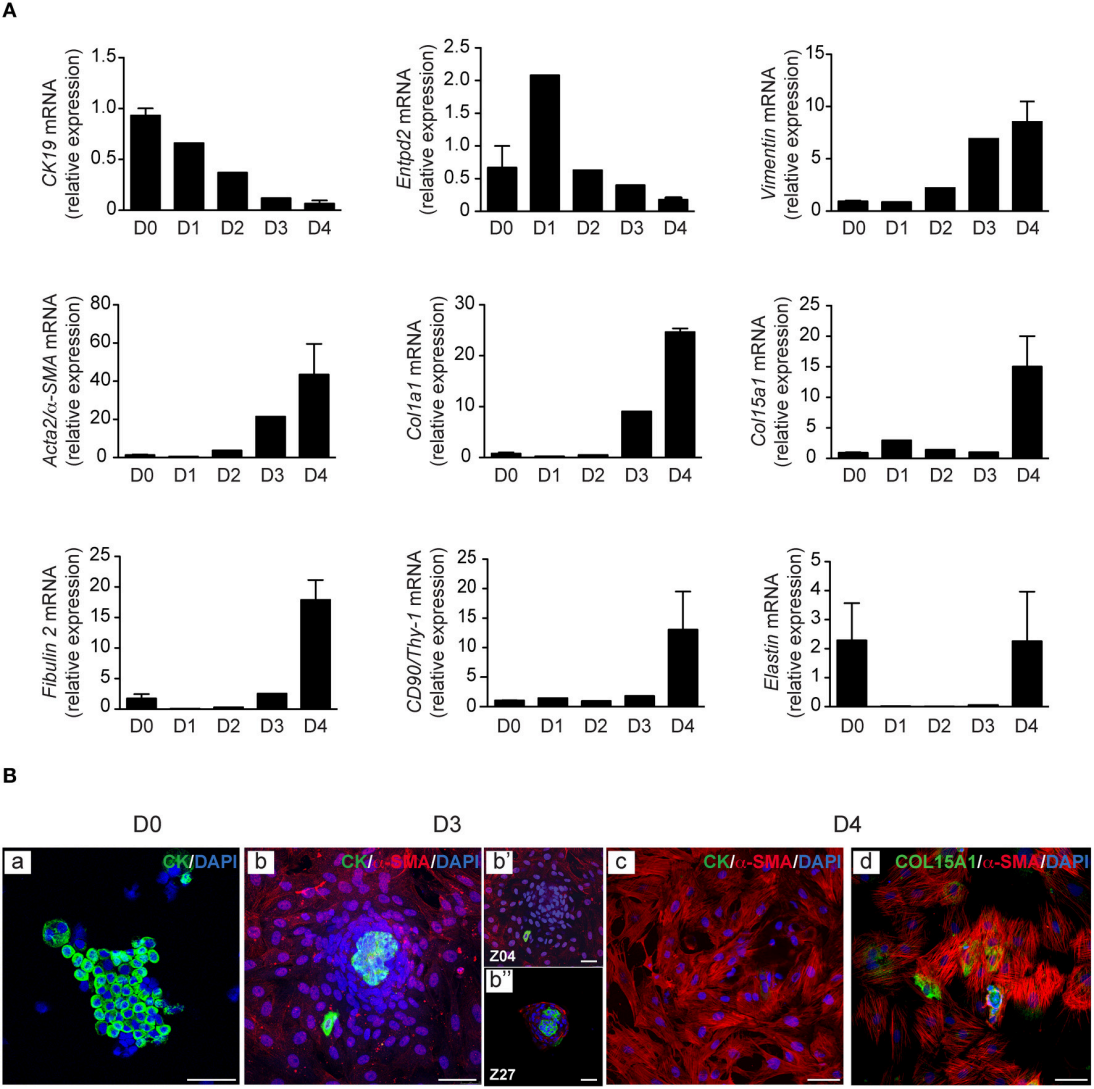
**Time course of cell markers in PMF culture**. PMF culture was subjected at different time points, to **(A)** RT-PCR analysis of cytokeratin 19 (*CK19*), ecto-nucleoside triphosphate diphosphohydrolase 2 (*Entpd2*), *Vimentin, Acta2/*α*-SMA, Col1a1, Col15a1, Fibulin 2, and CD90/Thy-1*; mRNA levels are normalized for *Hprt* and reported relative to those measured immediately after bile duct isolation (D0). They represent the means ± standard error of 2–3 preparations. **(B)** Immunofluorescence of cytokeratin (CK), and of α-SMA co-labeled with CK or COL15A1; nuclei were stained with DAPI; z images at 5.7 (Z04) and 13.6 (Z27) μm from the substrate are shown. D, days; Scale bars, 50 μm.

In the culture model of PMFs presented here, myofibroblasts are obtained by outgrowth from bile duct preparations. These preparations form small cell clusters that are entirely free of HSC contamination, as ascertained by the absence of vitamin A autofluorescence (Figure 1C). They initially contain a majority of bile duct epithelial cells as shown by cytokeratin staining (Figure 3Ba), and trace amounts of vascular structures as shown by α-SMA staining detectable in approximately one bile duct segment out of five (data not shown). They are placed in culture conditions with FBS, which contains factors such as TGF-β, that provide a growth advantage to mesenchymal cells over epithelial cells, in primary culture. In these conditions, bile duct epithelial cells die and detach from the substrate, while cells with a spindle-shape morphology, outgrow from the cell clusters. The time course of this evolution in culture can be observed by Video-Microscopy (Supplementary Video). Morphology of the cells observed under video-microscopy at different time points of culture, is shown in Figure 2. The time course of gene expression assessed by RT-PCR in the cultured cells (Figure 3A), shows that the expression of cytokeratin 19 (*CK19*) used as a marker of bile duct epithelial cells, progressively decreases over time, to become virtually absent at the stage of confluence, after 4 days in culture. The expression of *Entpd2*, previously shown to be a marker of portal fibroblasts and to undergo down-regulation during the myofibroblastic differentiation of these cells (Dranoff et al., 2002; Li et al., 2007; Wen et al., 2012), increases at day 1, when the fibroblastic cells emerge and decline thereafter. The expressions of *Vimentin*, a general marker of mesenchymal cells, and of *Acta2/*α*-SMA*, a marker of myofibroblasts, progressively increase after day 2. The expression of collagen, type I, alpha 1 (*Col1a1*) increases after day 3, whereas expressions of the PMF markers *Col15a1, Fibulin 2*, and *CD90/Thy-1*, are induced at the stage of confluence, after 4 days. We also found that the expression of elastin, a marker of portal fibroblasts, could be detected in bile duct isolates and in confluent PMFs, but not during the period of cell expansion in culture. In two independent studies of Collagen-α1(I)-green fluorescent protein (*Col1a1*^GFP^) mice with induced liver fibrosis, Fibulin 2, Thy-1 and elastin were overexpressed in cells considered as activated portal fibroblasts (Iwaisako et al., 2014; Lua et al., 2016). Consistent with RT-PCR analyses, dual immunolabeling shows that after 3 days, most of the cells are cytokeratin-negative (Figure 3Bb). Only a small number of cytokeratin-positive cells localized in the apex of the cell clusters are observed under confocal microscopy, whereas α-SMA is expressed in cells that adhere to the substrate (Figure 3Bbb'b”). Occasionally, a cytokeratin-positive cell adhering and migrating on the substrate, can be seen at the periphery of the cluster. However, we never detected co-expression of α-SMA and cytokeratin in the same cell, providing evidence against epithelial-mesenchymal transition in the emergence of PMF, although this mechanism could not be formally excluded. After 4 days, virtually all the cells are α-SMA-positive, whereas cytokeratin immunofluorescence is undetectable (Figure 3Bc). Co-expression of α-SMA and COL15A1 can be detected, although the intensity of COL15A1 immunofluorescence varies between individual cells (Figure 3Bd).

**Figure 4:**
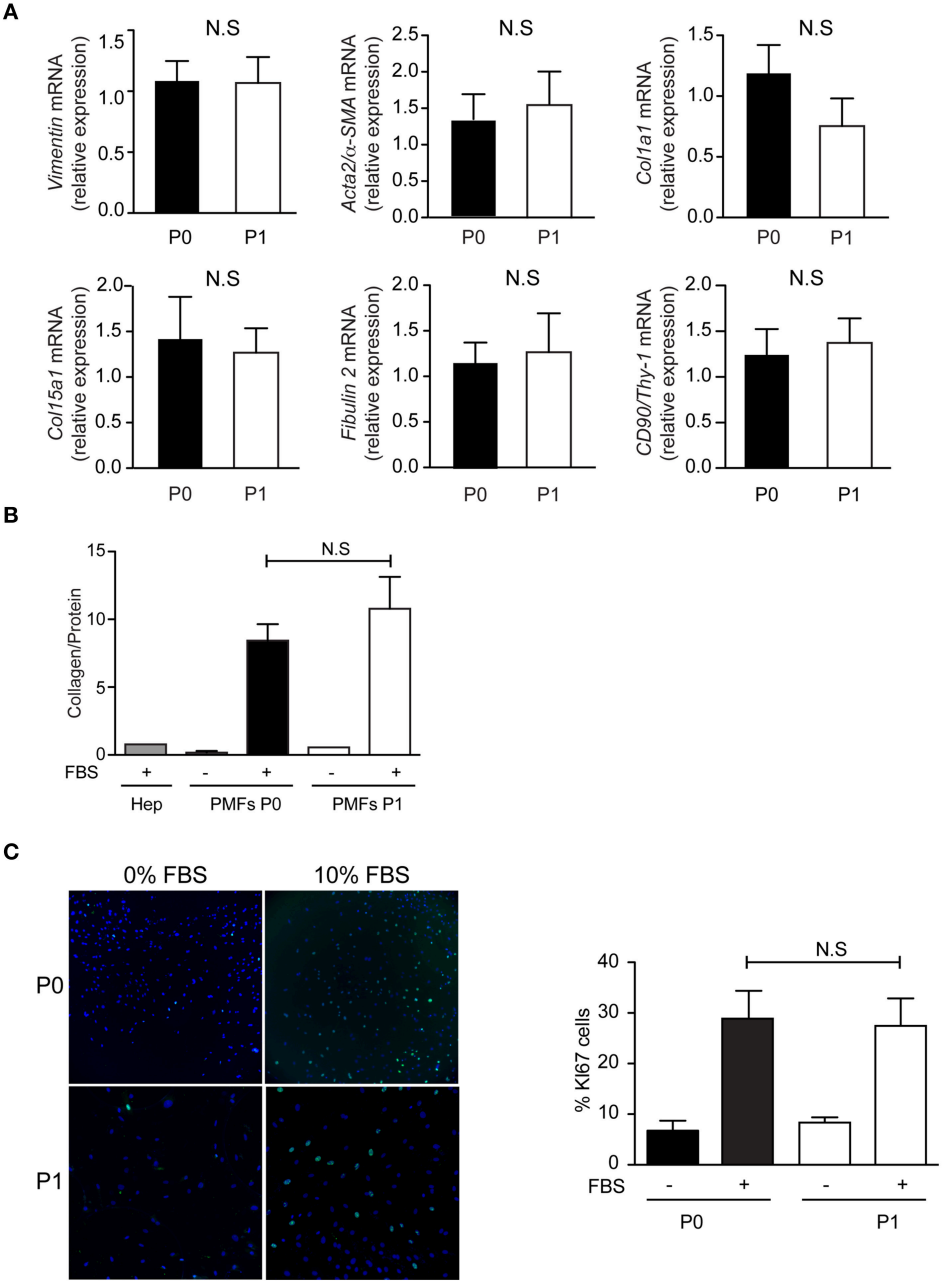
**Phenotype of PMFs in culture**. Characterization of PMFs at confluence in primary culture (P0) and after one passage (P1), by **(A)** RT-PCR analysis of *Vimentin, Acta2/*α*-SMA, Col1a1, Col15a1, Fibulin 2, and CD90/Thy-1*; mRNA levels are normalized for *Hprt* and reported relative to those measured at P0. They represent the means ± standard error of 7 preparations. **(B)** Collagen secretion, measured in the conditioned media of PMFs (*n* = 3–9) or hepatocytes (Hep, *n* = 1), used as controls, incubated with or without 10% FBS for the last 24 h; Results are normalized for total protein amount and represent the means ± standard error. **(C)** Proliferation assay by Ki67 immunofluorescence, in sub-confluent PMFs incubated with or without 10% FBS for the last 24 h; results are expressed as a percentage of Ki67-positive cells and represent the means ± standard error of 5–6 preparations. NS, not significant.

The comparison of confluent PMFs after 4–5 days, in primary culture (P0) and after one passage (P1), shows similar characteristics. The mRNA levels of *Vimentin, Acta2/*α*-SMA, Col1a1, Col15a1, Fibulin 2*, and *CD90/Thy-1*, are not significantly different between PMFs at P0 and P1 (Figure 4A). The mRNA levels of *Elastin* and *Entpd2* are not significantly different between P0 and P1 either (Data not shown), but too low to qualify them as PMF markers. Compared to serum-free conditions or primary rat hepatocytes, PMFs grown to confluence secrete collagen abundantly, without significant difference between P0 and P1 (Figure 4B). PMFs are also highly proliferative in the presence of serum, without significant difference between P0 and P1 (Figure 4C).

*In conclusion*, the protocol that we herein describe, provides a straightforward and reproducible method to obtain PMFs in culture. There is strong evidence to indicate that the progenitor cells giving rise to PMFs *in vivo*, are present in the initial bile duct isolates. These cells proliferate intensely, they acquire a fully differentiated myofibroblastic phenotype in primary culture and maintain this phenotype after one passage.

## Author contributions

HE: acquisition, analysis and interpretation of data, drafting and revision of the manuscript; EL: acquisition, analysis and interpretation of data, drafting and revision of the manuscript; SL: acquisition, analysis and interpretation of data, revision of the manuscript; AC: design and supervision of the work, acquisition, analysis and interpretation of data, drafting and revision of the manuscript; CH: conception and design of the work, interpretation of data, writing.

## Funding

EL received grant from the Fonds Cholangite Sclérosante Primitive.

### Conflict of interest statement

The authors declare that the research was conducted in the absence of any commercial or financial relationships that could be construed as a potential conflict of interest.

## Supplementary material

Video-Microscopy **PMF outgrowth in culture**. Bile duct isolates were plated in complete DMEM on u-Slide 2 Well Ph+ ibiTreat (Clinisciences), and placed at 37°C in a 5% CO_2_ incubator. A time-lapse video was acquired using phase contrast and 488 nm channels with a camera associated to an Olympus IX83 microscope at x20 magnification. This movie stands for a 76-h experiment (assembly of images of the same PMF culture, acquired from H0 to H36 and from H52 to H76).

## Abbreviations

α-SMA: alpha-smooth muscle actin
CK: cytokeratin
COL: collagen
D: day
ENTPD2: ectonucleoside triphosphate diphosphohydrolase-2
H: hour
Hep: hepatocyte
HPRT: hypoxanthine guanine phosphoribosyl transferase
HSC: Hepatic stellate cell
NS: not significant
PMF: portal myofibroblast.

## References

[B1] Beaussier M. Wendum D. Schiffer E. Dumont S. Rey C. Lienhart A.. (2007). Prominent contribution of portal mesenchymal cells to liver fibrosis in ischemic and obstructive cholestatic injuries. Lab. Invest. 87, 292–303. 10.1038/labinvest.3700513 17260005

[B2] Bosselut N. Housset C. Marcelo P. Rey C. Burmester T. Vinh J.. (2010). Distinct proteomic features of two fibrogenic liver cell populations: hepatic stellate cells and portal myofibroblasts. Proteomics 10, 1017–1028. 10.1002/pmic.200900257 20049859

[B3] Cassiman D. Libbrecht L. Desmet V. Denef C. Roskams T. (2002). Hepatic stellate cell/myofibroblast subpopulations in fibrotic human and rat livers. J. Hepatol. 36, 200–209. 10.1016/S0168-8278(01)00260-4 11830331

[B4] Dranoff J. A. Kruglov E. A. Robson S. C. Braun N. Zimmermann H. Sévigny J. (2002). The ecto-nucleoside triphosphate diphosphohydrolase NTPDase2/CD39L1 is expressed in a novel functional compartment within the liver. Hepatology 36, 1135–1144. 10.1053/jhep.2002.36823 12395323

[B5] Dudas J. Mansuroglu T. Batusic D. Ramadori G. (2009). Thy-1 is expressed in myofibroblasts but not found in hepatic stellate cells following liver injury. Histochem. Cell Biol. 131, 115–127. 10.1007/s00418-008-0503-y 18797914

[B6] Friedman S. L. (2008). Hepatic stellate cells: protean, multifunctional, and enigmatic cells of the liver. Physiol. Rev. 88, 125–172. 10.1152/physrev.00013.2007 18195085 PMC2888531

[B7] Hinz B. Phan S. H. Thannickal V. J. Prunotto M. Desmouliere A. Varga J.. (2012). Recent developments in myofibroblast biology: paradigms for connective tissue remodeling. Am. J. Pathol. 180, 1340–1355. 10.1016/j.ajpath.2012.02.004 22387320 PMC3640252

[B8] Iwaisako K. Jiang C. Zhang M. Cong M. Moore-Morris T. J. Park T. J.. (2014). Origin of myofibroblasts in the fibrotic liver in mice. Proc. Natl. Acad. Sci. U.S.A. 111, E3297–E3305. 10.1073/pnas.1400062111 25074909 PMC4136601

[B9] Kinnman N. Francoz C. Barbu V. Wendum D. Rey C. Hultcrantz R.. (2003). The myofibroblastic conversion of peribiliary fibrogenic cells distinct from hepatic stellate cells is stimulated by platelet-derived growth factor during liver fibrogenesis. Lab. Invest. 83, 163–173. 10.1097/01.LAB.0000054178.01162.E4 12594232

[B10] Knittel T. Kobold D. Saile B. Grundmann A. Neubauer K. Piscaglia F.. (1999). Rat liver myofibroblasts and hepatic stellate cells: different cell populations of the fibroblast lineage with fibrogenic potential. Gastroenterology 117, 1205–1221. 10.1016/S0016-5085(99)70407-5 10535885

[B11] Kruglov E. A. Jain D. Dranoff J. A. (2002). Isolation of primary rat liver fibroblasts. J. Investig. Med. 50, 179–184. 10.2310/6650.2002.33431 12033282

[B12] Lemoinne S. Cadoret A. El Mourabit H. Thabut D. Housset C. (2013). Origins and functions of liver myofibroblasts. Biochim. Biophys. Acta 1832, 948–954. 10.1016/j.bbadis.2013.02.019 23470555

[B13] Lemoinne S. Cadoret A. Rautou P. E. El Mourabit H. Ratziu V. Corpechot C.. (2015). Portal myofibroblasts promote vascular remodeling underlying cirrhosis formation through the release of microparticles. Hepatology 61, 1041–1055. 10.1002/hep.27318 25043701

[B14] Li Z. Dranoff J. A. Chan E. P. Uemura M. Sévigny J. Wells R. G. (2007). Transforming growth factor-beta and substrate stiffness regulate portal fibroblast activation in culture. Hepatology 46, 1246–1256. 10.1002/hep.21792 17625791

[B15] Lua I. Li Y. Zagory J. A. Wang K. S. French S. W. Sévigny J.. (2016). Characterization of hepatic stellate cells, portal fibroblasts, and mesothelial cells in normal and fibrotic livers. J. Hepatol. 10.1016/j.jhep.2016.01.010. [Epub ahead of print]. 26806818 PMC4834254

[B16] Mederacke I. Hsu C. C. Troeger J. S. Huebener P. Mu X. Dapito D. H.. (2013). Fate tracing reveals hepatic stellate cells as dominant contributors to liver fibrosis independent of its aetiology. Nat. Commun. 4, 2823. 10.1038/ncomms3823 24264436 PMC4059406

[B17] Ogawa T. Tateno C. Asahina K. Fujii H. Kawada N. Obara M.. (2007). Identification of vitamin A-free cells in a stellate cell-enriched fraction of normal rat liver as myofibroblasts. Histochem. Cell Biol. 127, 161–174. 10.1007/s00418-006-0237-7 17024455

[B18] Tuchweber B. Desmoulière A. Bochaton-Piallat M. L. Rubbia-Brandt L. Gabbiani G. (1996). Proliferation and phenotypic modulation of portal fibroblasts in the early stages of cholestatic fibrosis in the rat. Lab. Invest. 74, 265–278. 8569191

[B19] Uchio K. Tuchweber B. Manabe N. Gabbiani G. Rosenbaum J. Desmoulière A. (2002). Cellular retinol-binding protein-1 expression and modulation during *in vivo* and *in vitro* myofibroblastic differentiation of rat hepatic stellate cells and portal fibroblasts. Lab. Invest. 82, 619–628. 10.1038/labinvest.3780456 12004002

[B20] Wen J. W. Olsen A. L. Perepelyuk M. Wells R. G. (2012). Isolation of rat portal fibroblasts by *in situ* liver perfusion. J. Vis. Exp. 3669 10.3791/3669 PMC3476385 22781701

