# Supplementary material for: Culture Model of Rat Portal Myofibroblasts
Source: Front Physiol. 2016 Mar 31;7:120. doi: 10.3389/fphys.2016.00120 (PMC4814710; doi:10.3389/fphys.2016.00120)
Supplement: Supplementary file 2 [file Table1.DOCX]

**Supplemental table 1. Primers used for RT-PCR**

| **Gene** | **GenBank** | **Forward primer** | **Reverse primer** |
| --- | --- | --- | --- |
| *Acta2*  *CD90/Thy-1*  *CK19*  *Col1a1*  *Col15a1*  *Elastin*  *Entpd2*  *Fibulin 2*  *Hprt*  *Vimentin* | NM_031004.2  NM_012673.2  AY464140.1  NM_053304.1  NM_001100535.1  NM_012722.1  NM_172030.1  NC_005103  XM_008773659.1  NM_031140.1 | GGGACGACATGGAAAAGATCTG  CCACAAGCTCCAATAAAACTATCAA  ACGCGGTGGAAGTTTTAGTG  TGAGCCAGCAGATTGAGAAC  GCCCCCTACTTCATCCTCTC  GAGGAAAGCCTGGGAAAGTT  ACAGAGATCACCTGCAACCA  GAACCGCCAGTAGCAGGTAG  AGGACCTCTCGAAGTGT  AACACTCCTGATTAAGACGGTTG | GGTTGGCCTTAGGGTTCAGC  AGCAGCCAGGAAGTGTTTTG  CGAATCTTCACCTCCAGCTC  GGGTCGATCCAGTACTCTCCG  CAGTACGGACCTCCAGGGTA  GGATTCCAGAAAAAGCACCA  GGTCTTTTGTCCTGGCACTC  GCCTGACTTAGGCAGAGGTG  ATCCCTGAAGTGCTCATTATA  TCATCGTGGTGCTGAGAAGT |

*CK19*, cytokeratin 19; *Col1a1*, collagen, type I, alpha 1*; Col15a1*, collagen, type XV, alpha 1; *Entpd2*, ectonucleoside triphosphate diphosphohydrolase-2; *Hprt*, hypoxanthine guanine phosphoribosyl transferase.
